# Intravenous Glial Growth Factor 2 (GGF2) Isoform of Neuregulin-1β Improves Left Ventricular Function, Gene and Protein Expression in Rats after Myocardial Infarction

**DOI:** 10.1371/journal.pone.0055741

**Published:** 2013-02-21

**Authors:** Michael F. Hill, Amish V. Patel, Abigail Murphy, Holly M. Smith, Cristi L. Galindo, Laura Pentassuglia, Xuyang Peng, Carrie G. Lenneman, Oghenerukevwe Odiete, David B. Friedman, Marvin W. Kronenberg, Siyuen Zheng, Zhongming Zhao, Yanna Song, Frank E. Harrell, Maya Srinivas, Anindita Ganguly, Jennifer Iaci, Tom J. Parry, Anthony O. Caggiano, Douglas B. Sawyer

**Affiliations:** 1 Division of Cardiovascular Medicine, Department of Medicine, Vanderbilt University Medical Center, Nashville, Tennessee, United States of America; 2 Department of Biochemistry, Vanderbilt University Medical Center, Nashville, Tennessee, United States of America; 3 Biomedical Informatics and Cancer Biology, Vanderbilt University Medical Center, Nashville, Tennessee, United States of America; 4 Department of Biostatistics, Vanderbilt University Medical Center, Nashville, Tennessee, United States of America; 5 Acorda Therapeutics, Inc., Hawthorne, New York, United States of America; University of Medicine and Dentistry of New Jersey, New Jersey Medical School, United States of America

## Abstract

**Aims:**

Recombinant Neuregulin (NRG)-1β has multiple beneficial effects on cardiac myocytes in culture, and has potential as a clinical therapy for heart failure (HF). A number of factors may influence the effect of NRG-1β on cardiac function via ErbB receptor coupling and expression. We examined the effect of the NRG-1β isoform, glial growth factor 2 (GGF2), in rats with myocardial infarction (MI) and determined the impact of high-fat diet as well as chronicity of disease on GGF2 induced improvement in left ventricular systolic function. Potential mechanisms for GGF2 effects on the remote myocardium were explored using microarray and proteomic analysis.

**Methods and Results:**

Rats with MI were randomized to receive vehicle, 0.625 mg/kg, or 3.25 mg/kg GGF2 in the presence and absence of high-fat feeding beginning at day 7 post-MI and continuing for 4 weeks. Residual left ventricular (LV) function was improved in both of the GGF2 treatment groups compared with the vehicle treated MI group at 4 weeks of treatment as assessed by echocardiography. High-fat diet did not prevent the effects of high dose GGF2. In experiments where treatment was delayed until 8 weeks after MI, high but not low dose GGF2 treatment was associated with improved systolic function. mRNA and protein expression analysis of remote left ventricular tissue revealed a number of changes in myocardial gene and protein expression altered by MI that were normalized by GGF2 treatment, many of which are involved in energy production.

**Conclusions:**

This study demonstrates that in rats with MI induced systolic dysfunction, GGF2 treatment improves cardiac function. There are differences in sensitivity of the myocardium to GGF2 effects when administered early vs. late post-MI that may be important to consider in the development of GGF2 in humans.

## Introduction

Congestive heart failure (CHF) subsequent to myocardial infarction (MI) and other forms of cardiac injury are common clinical problems with a poor prognosis [1]. Cardiac dysfunction is frequently characterized at the cellular level by abnormalities in the calcium handling, metabolism, sarcomere synthesis and survival of cardiac myocytes. Animal studies have shown that neuregulin-1β (NRG-1β) is a critical regulator of these processes. NRG-1β is released from cardiac microvascular endothelial cells and acts as a paracrine factor via the ErbB family of tyrosine kinase receptors expressed in cardiac myocytes to regulate myocyte differentiation and stress responses [2], [3]. Enhancement of NRG-1β signaling via administration of recombinant fragment of NRG-1β improves cardiac systolic function as well as survival in animal models of ischemic, dilated, and viral cardiomyopathy [4]. Clinical trials with a small fragment of NRG-1β suggest positive effects on cardiac function of CHF patients [5], [6]. Collectively, these results suggest that NRG-1β may be a promising therapeutic agent for CHF.

The biologic effects of NRG-1β are mediated through ErbB2, 3 and 4 receptor tyrosine kinases, where ErbB3 and 4 bind NRGs directly, and ErbB2 acts as a heterodimerization partner. The activity of NRG-1β will therefore vary with conditions that alter ErbB receptor expression or activity. In cultured myocytes, for example, antibodies to ErbB2 receptors alter cellular response to NRG-1β [7], [8], [9], [10]. ErbB2 receptors are HSP90 client proteins, and their stability is a function of metabolic stress. Hence, in the setting of small perturbations of ATP/ADP ratios, there is degradation of ErbB2 and reduced responsiveness to NRG-1β [11]. Furthermore, coupling of ErbB2 and ErbB4 receptors is markedly altered by the presence of saturated fatty acids, at least in isolated cardiac myocytes [12]. This last observation raises the question of whether the beneficial effects of recombinant NRG-1β may be suppressed, or even reversed, by high dietary fat.

One of the multiple isoforms of NRG-1β is the kringle-containing form originally named for its mitogenic effects on glial cells as glial growth factor 2 (GGF2) [13]. GGF2 has high potency in isolated cardiac myocytes [14], and may offer advantages over other isoforms in that the kringle domain has matrix binding properties that could potentiate its biological half-life in tissues. GGF2 has been shown to improve cardiac function in post-MI rats [15]. The primary objective of this study was to determine if the beneficial effects of GGF2 on post-MI cardiac function varied under conditions of high-fat feeding or timing of treatment. A secondary objective was to examine potential mechanisms for these effects of GGF2 through genomic and proteomic analysis of the myocardium.

## Methods

### Experimental Animals

Sprague Dawley rats were obtained from Charles River Laboratories (Wilmington, MA). Experiments were performed according to a protocol approved by the Institutional Animal Care and Use Committee at Vanderbilt University Medical Center (Protocol ID#: M/09/269). The investigation conforms to the *Guide for the Care and Use of Laboratory Animals* published by the US National Institutes of Health and the standards of the Association for Assessment and Accreditation of Laboratory Animal Care. All surgeries were performed under anesthesia as detailed below. Every animal received post-operative pain management, and all efforts were made to minimize suffering.

### Induction of Myocardial Infarction (MI)

Male Sprague Dawley rats (175–200 g body weight) were anaesthetized intraperitoneally with 30 mg/kg of pentobarbital. Absence of a response to pinching the toe was used as an indicator of the appropriate level of anesthesia. Rats were then rapidly intubated and mechanically ventilated (tidal volume, 1 mL/100 g body weight; ventilation rate, 65 strokes/min) by a constant volume small animal ventilator (Model 683, Harvard Apparatus). A left thoracotomy was performed at the fourth intercostal space, and the left coronary artery was then ligated by irreversible tightening of a 6–0 suture loop. MI was confirmed by regional cyanosis of the myocardial surface distal to the suture, accompanied by S-T segment elevation on the electrocardiogram. Following successful induction of MI, the chest cavity was compressed to evacuate any air before being tightly sealed. Sham-operated animals underwent the same surgical procedure with the exception that the left coronary artery was not ligated. The rats were given buprenorphine 0.05 mg/kg post-operatively, then Q 8–12 hours post-operatively by subcutaneous injection. The rats that survived through day 7 post-MI were randomly assigned to various groups as outlined in the experimental design.

### Diet

All rats were maintained on a normal chow diet of 12% calories from fat, 28% calories from protein, and 60% calories from carbohydrate (LabDiet) prior to coronary ligation surgery. Following surgery, rats in the study examining effects of GGF2 early after MI were also randomly assigned to either a normal diet or a high saturated fat diet of 43.5% calories from fat, 22.2% calories from protein, and 34.3% calories from carbohydrate (LabDiet) beginning 7 days post-MI and continuing until completion of the study. All rats in the study examining effects of GGF2 late after MI were continuously maintained on a normal chow diet.

### GGF2 Treatment

Recombinant GGF2 (96.0% purity by SEC-HPLC) was provided by Acorda Therapeutics, Inc. and stored at 4°C. GGF2 was used at low dose (0.625 mg/kg) or high dose (3.25 mg/kg) based upon rat weights taken at each day of treatment. GGF2 was administered via the right lateral tail vein every second day to surviving MI rats starting one or 8 weeks after MI. Vehicle-treated rats received equal volume injections of sterile GGF2 diluent (20 mM histidine, 100 mM arginine, 100 mM sodium sulfate, 1% mannitol, pH 6.5).

### Positron Emission Tomographic (PET) Imaging

Rats in the study examining effects of GGF2 late after MI underwent microPET imaging to evaluate myocardial metabolic changes and myocardial viability post-infarction. Anesthesia was induced using 2–3% isoflurane/97–98% oxygen supplied via nosecone. Fasting rats were administered an intraperitoneal injection of ^18^F-fluorodeoxyglucose (^18^FDG) and anesthesia was discontinued. After a sixty minute rest period to allow for uptake of radioisotope tracer into tissues, anesthesia was re-induced for the imaging procedure. A small-animal tomograph (Siemens MicroPET Focus 220) acquired full body PET images for approximately 30–45 minutes. Images were later analyzed with Amide software. Mean cardiac standard uptake values (SUVs) were calculated from transverse slices (0.47 mm thickness) by drawing 3-dimensional, free hand regions of interest that completely encircled the heart. Regions of interest approximately 0.5 cm in diameter were also drawn on each animal's right shoulder skeletal muscle. Mean cardiac SUVs were then normalized to shoulder muscle SUVs. After completing the PET procedure, animals were placed in a recovery cage and monitored until fully ambulatory. Rats were scanned at 8, 10, and 12 weeks post-MI. All scanning and interpretation was performed blinded to the treatment group.

### Assessment of Cardiac Function by Echocardiography

Transthoracic echocardiographic images of hearts from all groups of rats were obtained using a 12-MHz ultrasound probe (model s12, Agilent Technologies) and an echograph (SONOS 5500, Hewlett Packard) while rats were immobilized under anesthesia (1.5–2.0% isoflurane). For M-mode recordings, the parasternal short-axis view was used to image the heart in two dimensions at the level of the papillary muscles. End-diastolic and end-systolic LV cavity dimensions were measured using software resident on the ultrasonograph. LV fractional shortening (FS) was calculated from M-mode-derived left ventricular inner diameters in diastole (LVIDd) and systole (LVIDs) using the formula (LVIDd – LVIDs)/LVIDd x100%. All imaging and interpretation of results was done blinded to treatment group. In all studies rats underwent serial echocardiograms at baseline prior to initiation of treatment, and subsequently at two week intervals until sacrifice.

### Histology

All histological evaluation was performed in a blinded fashion. Paraffin-embedded sections (4 µm) of hearts from all of the groups were stained with Masson's trichome. Cardiac fibrosis in the LV remote from the site of infarction was measured in using NIH ImageJ software and was expressed in arbitrary units as a percentage of the total area of the tissue section stained.

### Measurement of Myocardial Oxidative Stress

We examined myocardial oxidative stress by measuring myocardial protein carbonylation as previously described [16] using OxyBlot protein oxidation detection kit (Millipore) according to manufacturers instructions. Tssue lysates were prepared from non-infarcted region of the left ventricle representing 15 µg of protein. Blots were developed using fast tablet (BCIP/NBT; Sigma-Aldrich) and quantified using Scion Image (PC version of Macintosh-compatible NIH Image) software. No non-specific background binding of the primary or secondary antibodies was found.

### Western Blot Analysis of ErbB2 and ErbB4 Expression

Remote, non-infarct left ventricular tissue from all groups was homogenized using a liquid nitrogen chilled mortar and pestle, in a liquid nitrogen chilled CryoGrinder. Homogenization was performed using RIPA buffer (Millipore), supplemented with protease inhibitor cocktail (Roche). Proteins were quantified with Bradford assay (Bio-Rad). 50 micrograms of protein in SDS sample buffer was heated using a Dry Bath incubator (Fisher scientific) for 10 minutes and after centrifugation, loaded onto 4–12% NuPage gels (Invitrogen). After electrophoresis, proteins were transferred to an immunoblot PVDF membrane, using the Semi-Dry transfer apparatus at 20 V for 30 mins. Membranes were blocked using PBST-5% BSA and then incubated overnight at 4°C with antibodies directed against ErbB2 and ErbB4 (clone F-11 and clone C-18 respectively, Santa Cruz Biotechnology), each diluted 1∶500. Secondary horseradish peroxidase-conjugated antibody (Cell Signaling) was applied for 1 hour at room temperature. Blots were visualized using the SuperSignal West Pico chemiluminiscent substrate (Pierce) and analyzed with NIH ImageJ densitometry software.

### Transcriptome Analysis

Total RNA was extracted from the non-infarcted LV using Trizol (Invitrogen Co.) and RNeasy columns (Qiagen, Valencia, CA), according to the manufacturers' instructions, followed by DNAse treatment (Qiagen). RNA quality measurements using a 2100 Bioanalyzer (Agilent, Santa Clara, CA) and further sample processing were performed in the Genome Sciences Resource (GSR) Core at Vanderbilt. Processed and labeled samples were subsequently hybridized to the rat Gene 1.0 ST whole-transcriptome array (Affymetrix, Santa Clara, CA). Three biological replicates were used for each sample type as follows: Sham-operated, early MI rats (vehicle and low dose [0.625 mg/kg] GGF2-treated), and late MI rats (vehicle and high dose [3.25 mg/kg] GGF2-treated). Therefore, there were a total of 15 samples (5 sample types, n = 3). Only rats on the standard diet were chosen for transcriptome analysis, due to minimal differences in GGF2-mediated responses in rats fed a high versus low-fat diet.

Subsequent to import of raw data (.CEL) files, Robust Multi-chip Average (RMA) normalization [17] followed by statistical analysis was performed using Partek Genomics Suite version 6.6 (Partek Incorporated, St. Louis, MO). Quality assessment was performed based on Affymetrix internal controls, Box-and-Whisker plots, histograms, PCA (principal components analysis), and standard deviation of biological replicates. Based on these criteria, one sample (one of the low-dose, early GGF2-treated biological replicates) was identified as an outlier. We were unable to determine the source of the variation, which could have been technical or biologically relevant, and therefore performed analyses with and without the outlier sample. Following normalization and quality assessment, Partek was used to perform pairwise comparisons of average group values and one-way ANOVA. Only transcripts that resulted in a fold-change of at least 1.5 and p value of less than 0.05 were considered as significantly altered.

Gene functions, as presented in significant gene list tables, were determined using publicly available records using NCBI Entrez Gene, Stanford SOURCE, Aceview, and Pubmed databases. Statistical analyses (including correction for multiple hypothesis testing) for identification of overrepresented ontologies and functions were performed using Ingenuity Pathway Analysis (IPA) software (ingenuity Systems, Redwood City, CA).

For comparison of our gene expression results to previous studies, raw microarray data (CEL or text files) from four rat MI studies were downloaded from Gene Expression Omnibus (GEO) (http://www.ncbi.nlm.nih.gov/geo/). Gene expression data from autopsied human hearts and failing hearts collected during heart transplant surgeries were also obtained from ArrayExpress Archive database (www.ebi.ac.uk/arrayexpress) [18] under accession number E-TABM-480 [19], and the GEO database under accession numbers GSE1145 [20], GSE1869 [20], and GSE5406 [21]. Combined, our study, the four other rat studies, and the human studies included 363 individual biological samples. Raw data from other studies were processed similarly to ours, including RMA normalization using Partek Genomics Suite and fold-change of 1.5 or greater and p value ≤0.05 considered as altered, to make the various studies more comparable.

To link our gene expression results (i.e., gene expression profiles of GGF2-treated early and late MI rats) to results obtained from the various other animal and human studies, a perl script was written to match genes across the various lists by accession number (for same species results) or gene symbol (for inter-species results comparisons). If neither the accession number nor gene symbol matched perfectly, then the gene descriptions were compared and manually checked if the difference did not exceed 25%. If a data set contained more than one match for a single data point in a published study, only the top three matches were recorded, with the best match ordered first. This perl script was later modified to compute averages for the multiple matches. This method would be expected to fail to identify some homologous genes in which there are name designation differences between species (e.g., the mouse version of human IL-8 is referred to as KC or GRO). However, our intention was to greatly minimize false positives and thus compare only those genes that were truly the same between the various data sets. To ensure that this was indeed the case, all final gene lists representing overlap between study results were also manually examined and any genes that were computationally misidentified were subsequently removed.

### Real-time RT-PCR (qRT-PCR)

Real-time RT-PCR was performed to quantify transcript levels for a subset of genes using the CFX96 C-1000 (Bio-Rad, Hercules, CA) using SYBR Green I dye (QIAGEN), per manufacturer's instructions. Briefly, each 25-μl reaction contained 100 ng of RNA, 2.5 μl of primers (Quantitect Primer Assays; QIAGEN), 12.5 μl of SYBR Green PCR master mix and 0.25 μl of reverse transcriptase. Negative controls containing water instead of RNA were concomitantly run to confirm that the samples were not cross-contaminated. Targets were normalized to reactions performed using Quantitect TPT1 primers (QIAGEN), which amplify a translationally-controlled transcript. Fold change was determined using the comparative threshold method [22].

### Difference Gel Electrophoresis and Differential-Display Proteome Analysis

Myocardial tissue originating from the remote, viable LV in all groups of rats was immediately frozen in liquid nitrogen and stored at –80°C until use. Frozen viable LV tissues were homogenized in lysis buffer consisting of 7 M urea, 2 M thiourea, 30 mM Tris, 5 mM magnesium acetate, 4% CHAPS, and 58 mM DTT using a ratio of 1 g tissue:10 ml lysis buffer. The lysate was centrifuged at 12,000×g for 1 h at 10°C. The pellet was discarded and the protein concentration of the supernatant was determined using a 2-D Quant protein assay kit (Amersham Biosciences/GE Healthcare, Piscataway, New Jersey).

250 µg from each of the samples were precipitated with methanol/chloroform [23], [24] and resuspended in 40 µL labeling buffer (7 M urea, 2 M thiourea, 4% CHAPS, 30 mM Tris, 5 mM magnesium acetate). One-third of each sample (83 µg) was removed and pooled into a separate tube for the mixed-sample internal standard. The remaining 167 µg of each sample was separately labeled with 200 pmoles of either Cy3 or Cy5 NHS-ester minimal labeling reagents (2 µL of a 100 pmol/µL in NN di-methyl foramide) for 30 min on ice in the dark, followed by quenching with 2 µL of 10 mM lysine for 10 min on ice in the dark. The pooled sample mixture (830 µg total) was similarly labeled with 1,000 pmol of Cy2 and quenched with 10 µL of 10 mM lysine. Individual Cy3- and Cy5-labeled samples were combined with an equal portion of the Cy2-labeled internal standard for a total of approximately 300 µg on each gel.

All equipment was manufactured by GE Healthcare/Amersham Biosciences (Piscataway, New Jersey) unless otherwise noted. The samples were separated by standard 2D gel electrophoresis using a manifold-equipped IPGphor first-dimension isoelectric focusing unit and 24 cm 4–7 immobilized pH gradient (IPG) strips, followed by second-dimension 12% SDS-PAGE homogenous on hand-cast gels that had one plate pre-silanized to ensure accurate robot picking in subsequent steps, using an Ettan DALT 12 unit according to the manufacturer's protocols. The Cy2 (mixed standard), Cy3 (sample X) and Cy5 (sample Y) components of each gel were individually imaged at 100 µm resolution with mutually-exclusive excitation/emission wavelengths using a Typhoon 9400 Variable Mode Fluorescence Imager. A Sypro Ruby total protein post-stain (Invitrogen/Molecular Probes) was used to ensure accurate protein excision, as the low stoichiometry of Cy-dyes label only 1–3% of the total protein.

DeCyder software v6.5 was used for simultaneous comparison of abundance changes across all sample pairs with statistical confidence and without interference from gel-to-gel variation [25], [26]. Normalized volume ratios were calculated for each resolved protein on every gel, and the internal standard signals were used to normalize and compare these ratios across all five gels to allow for the calculation of average abundance changes and p-values for the variance of these ratios for each protein-pair across all five independent gels.

### Mass Spectrometry and Database Interrogation

Proteins of interest were robotically excised, digested into peptides in-gel with modified porcine trypsin protease (Trypsin Gold, Promega) and peptides extracted using the automated Ettan Spot Handling Workstation (GE Healthcare) using 20 μL 20 mM NH_4_HCO_3_ containing 0.01 μg/μL Trypsin Gold (Promega) for 3 h at 37°C for protein digestion. Peptides were extracted by two rounds of incubation with 60% acetonitrile, 0.1% trifluoroacetic acid, dried and reconstituted in 15 μL 0.1% formic acid and placed into autosampler vials. 5 μL of each peptide hydrosylate was separately analyzed by C18 reverse-phase LC-MS/MS using a Thermo LTQ ion trap mass spectrometer equipped with a Thermo MicroAS autosampler and Eksigent HPLC nanoLC pump system, nanospray source, and Xcalibur 2.0 instrument control using standard data-dependent methods. Tandem MS data were analyzed with the Sequest algorithm, searching the IPI_rat-v342 database (Apr 2008) that contained a concatenated reverse decoy database to estimate false-discovery rates. Search results were filtered by cross-correlation scores (<1.0 for singly-charged peptides, <1.8 for doubly-charged, and <2.5 for triply-charged) with an overall 2.4% false-discovery rate. Protein identifications were based on a minimum of 2 peptides passing these criteria for each protein. The number of unique peptides and the total number of peptide identifications (spectral counts) for each protein are listed, and the predicted MW and pI were correlated with the relative gel position from which the protein was excised.

### Statistical Analysis

Gene expression and real-time RT-PCR statistical analyses were performed as described above. All other statistical analyses were completed using statistical software R version 2.13.0 (2011–04–13). Echocardiography data were analyzed by using the generalized least squares model. This linear regression model accounts for correlation across time within animals and was fit to investigate whether GGF2 treatment and diet affected the 35-day outcome (FS %). Estimated mean values were fit using restricted maximum likelihood or REML and are presented with its 95% confidence interval. Non- linearity was addressed by applying a restricted cubic spline term, and transformation was applied when the normality assumption did not hold. Time by treatment and time by diet interactions were added to the model, and Wald statistics were used to assess the individual and joint significance of regression coefficients. Other data were analyzed by ordinary least squares and one-way ANOVA as indicated.

## Results

### GGF2 Therapy Improves Cardiac Function Following Recent MI

In total, 103 rats underwent surgery, with 98 rats receiving coronary artery ligation (MI) and 5 rats receiving sham operation. Overall survival rate among the MI rats was 87.8%. All of the sham-operated rats survived. This resulted in 86 MI and 5 sham-operated animals that were included in the present study. No deleterious side effects were observed in any of the GGF2-treated rats throughout the treatment periods.

**Figure 1:**
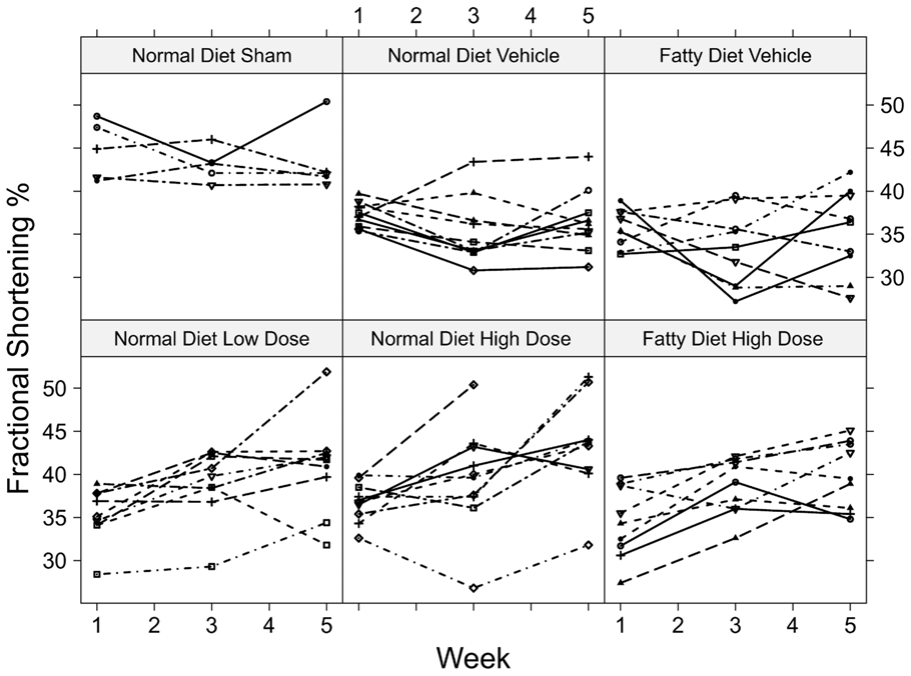
Effect of GGF2 treatment on cardiac function early after MI. Serial measurements of fractional shortening (FS) were acquired from post-MI rats at baseline (1 week post-MI), 2 weeks after GGF2 treatment, and 4 weeks after GGF2 treatment. Echocardiographic assessment revealed that residual left ventricular (LV) FS% values were significantly higher (p = 0.0001) in GGF2 treated animals compared to those in the vehicle groups at the end of the study. The estimated mean FS% and range at 35 days post MI was 43.6 (41.0, 46.3) for the high dose treated group (n = 9), 42.0 (39.1, 45.0) for the low dose treated group (n = 9), and 36.6 (34.2, 39.1) for the vehicle group (n = 10. Individual rat FS % values trended downwards in the vehicle animals with time, whereas a progressive increase in FS % was observed in both the low dose and high dose GGF2 treatment groups. Moreover, the results indicate that high-fat feeding did not impair the effects of high dose GGF2 treatment on cardiac function.

In the first group of rats, the effects of two doses of intravenous GGF2 with treatment started 7 days after MI were compared. Rats were randomly assigned to treatment groups. Echocardiographic assessment of left ventricular systolic function one week after MI or sham operation showed similar cardiac dysfunction in all groups compared to sham-operated rats. FS% was improved in GGF2 treated groups compared to those in the vehicle treated groups by the end of 4 weeks treatment (Figure 1, Table 1). Comparison of low dose vs. high dose treated rats at 2 and 4 weeks of GGF2 administration showed that there was no significant difference in FS values (p = 0.70, p = 0.82 respectively). Thus, both doses of GGF2 studied improved cardiac contractile function in rats with recent MI-induced systolic dysfunction.

**Table 1:** Echocardiographic Measurements and Tissue Weight Ratios.

Group	Week	FS %^†^	P Value^†^	LVIDd^‡^	Heart/Body^§^
Normal Diet, Vehicle	1	36.5		0.75	
	3	34.7 (32.2, 37.1)	NS	0.77 (0.74, 0.81)	
	5	36.6 (34.2, 39.1)	NS	0.79 (0.75, 0.82)	1.42 (1.29,1.57)
Low Dose GGF2	1	36.5		0.75	
	3	40.1 (37.1, 43.1)	NS	0.74 (0.71, 0.78)	
	5	42.0 (39.1, 45.0)	0.005*	0.76 (0.72, 0.80)	1.27 (1.13, 1.43)
High Dose GGF2	1	36.5		0.75	
	3	40.3 (37.7, 42.9)	NS	0.76 (0.73, 0.80)	
	5	43.6 (41.0, 46.3)	0.0001*	0.77 (0.73, 0.80)	1.49 (1.34, 1.65)
Fatty Diet, Vehicle	1	36.5		0.75	
	3	34.6 (32.0, 37.2)	NS	0.74 (0.71, 0.78)	
	5	35.6 (33.0, 38.2)	NS	0.75 (0.71, 0.78)	1.38 (1.25,1.53)
High Dose GGF2	1	36.5		0.75	
	3	40.2 (37.3, 43.1)	NS	0.73 (0.70, 0.77)	
	5	42.6 (39.7, 45.4)	0.0001*	0.73 (0.69, 0.77)	1.44 (1.30,1.60)

*FS%* fractional shortening, *LVIDd* left ventricular inner diameter at diastole, and *Heart/Body* heart weight to body weight ratio. Values are shown as predicted means with its 95% confidence interval. Groups are stratified by treatment and diet as follows: Normal Diet-Vehicle (n = 10), Normal Diet-Low Dose GGF2 (n = 9), Normal Diet-High Dose GGF2 (n = 9), Fatty Diet-Vehicle (n = 9), and Fatty Diet-High Dose GGF2 (n = 9). ^†^FS % changes over time were analyzed using the generalized least squares linear regression method, and estimated means were fit using restricted maximum likelihood. Week 1 pre-treatment values were adjusted by adding a restricted cubic spline to the model. *Indicates significance with p<0.05 compared to vehicle animals. ^‡^LVIDd changes over time were also analyzed using GLS regression fit by REML, and week 1 pre-treatment values were adjusted by restricted cubic spline. No significant difference was found between groups at each time point. ^§^Heart/Body weight ratios were analyzed using ordinary least squares and one-way ANOVA. Heart/Body weight ratio comparison was found to be non-significant between groups.

### High-fat Diet Post-MI Does Not Alter GGF2's-Effect on Cardiac Function

To evaluate the potential effects of diet on GGF2 cardiac function, some animals were randomized to high fat diet vehicle and high fat diet GGF2-treated groups beginning 7 days post-MI and continuing until the end of the study. Results indicated that fatty diet had no significant influence on FS% values (p = 0.77) compared to normal diet, similar to previous reports [27], [28]. Specifically, comparison of FS% in high fat vs. normal diet rats at 2 and 4 weeks of high dose GGF2 administration were not significantly different (p = 0.72, p = 0.83 respectively) (Figure 1, Table 1). The total interaction of time by diet and time by treatment was found to be non-significant (p = 0.76), concluding that the observed treatment effect was not dependent on explanatory variables (Table 1). In addition, a high-fat diet did not alter LV dimensions in either the GGF2 treatment groups or the un-treated MI group of rats, compared with animals that were fed a low-fat diet (Table 1).

### Reduced Sensitivity to GGF2 Treatment Late After MI

**Figure 2:**
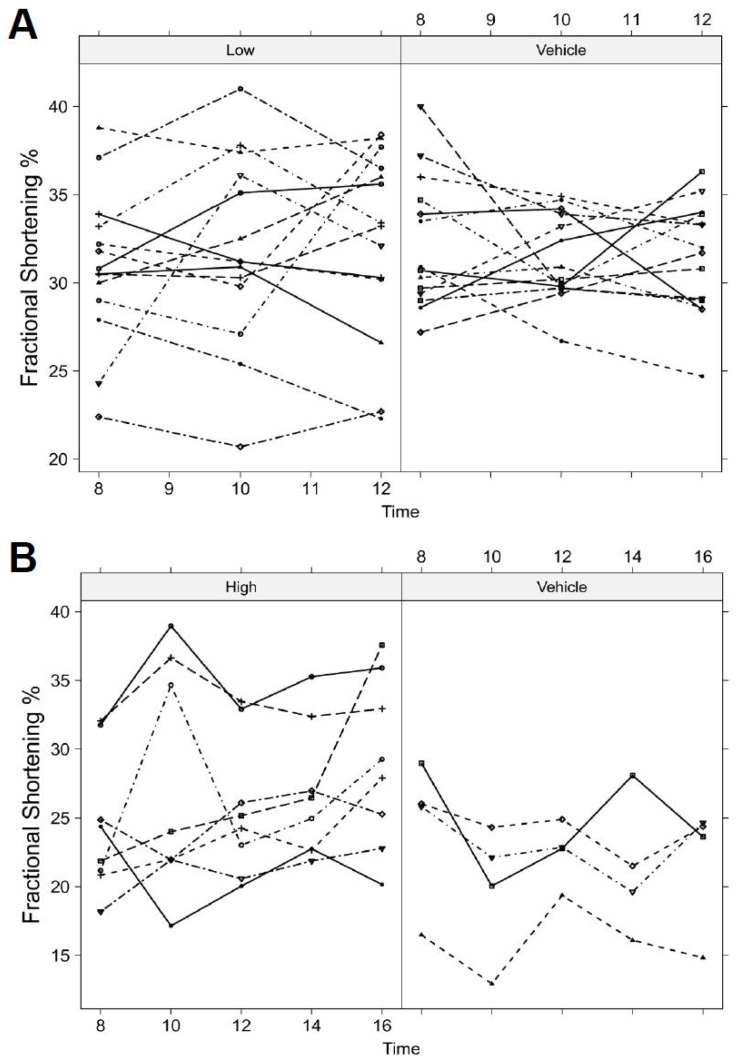
Effect of GGF2 treatment on cardiac function late after MI. A) Low dose GGF2: Rat FS % values were progressively reduced in both vehicle (n = 14) and low dose GGF-2 treated (n = 14) animals with time. The estimated mean FS% and range at 12 weeks post-MI was 32.9 (30.5, 35.3) for the low dose treated group and 31.7 (29.4, 33.9) for the vehicle group. Overall, the low dose treatment regimen did not restore late-post infarct LV structure and function because the average treatment effect was statistically insignificant (p = 0.51). B) High dose GGF2: FS % values decreased in vehicle (n = 4) animals and rose in high dose GGF2 treated (n = 8) rats with time. FS % was significantly increased in the high dose treated animals at 10,12,14, and 16 weeks post-MI (p = 0.013, p = 0.002, p = 0.002, and p = 0.017 respectively.) The estimated mean FS% and range at 16 weeks post-MI was 27.8 (24.1, 32.0) for the high dose treated group and 21.7 (18.1, 26.0) for the vehicle group. The average treatment effect of high dose GGF2 was found to be statistically significant (p = 0.005).

We examined the effects of GGF2 treatment on LV function and remodeling late after MI in rats. The late-infarct model underwent the same MI procedure; however, the rats were left untreated for 8 weeks prior to starting GGF2 or vehicle treatment. The first delayed treatment rats received intravenous low dose GGF2 (0.625 mg/kg) or vehicle three times per week. Transthoracic echocardiography was used to evaluate LV function at 8 weeks post-MI (pre-treatment) and after 2 and 4 weeks of treatment. FS% values were not significantly higher (p = 0.27) in low dose GGF2 treated compared to the vehicle group by the end of study (Figure 2A).

**Figure 3:**
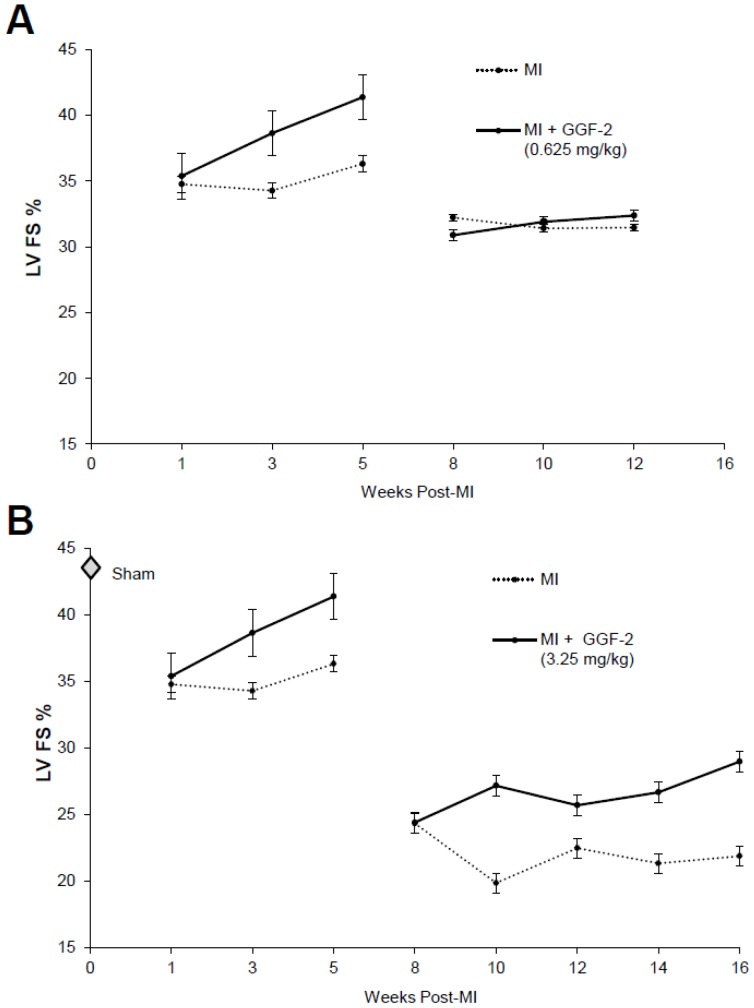
Comparison of effects of GGF2 in rats early and late after MI. Data from Figures 1 and 2 are shown for direct comparison of effects of A) low dose and B) high dose GGF2 (mean +/− S.D.).

We therefore repeated the experiment using a higher dose of GGF2 (3.25 mg/kg) in a new cohort of rats with initiation of therapy at 8 weeks post-MI. We further extended the treatment period by another 4 weeks (8 weeks total). In contrast to low dose GGF2 treatment late after MI, we found that FS % was significantly improved in high dose GGF2 treated animals compared to the vehicle group at 10,12,14, and 16 weeks post-MI (p = 0.013, p = 0.0016, p = 0.0018, and p = 0.017 respectively) (Figure 2B). Time by treatment interaction was found to be non-significant by Wald test (p = 0.95). The average treatment effect of high dose GGF2 in rats late after MI resulted in significantly improved FS% (p = 0.0046), while the average treatment effect of low dose GGF2 showed no difference from vehicle animals (p = 0.51). The dose-dependency between early and late post-MI treatment is further illustrated in Figures 3A and 3B.

### Analysis of Myocardial Viability and Metabolism

**Figure 4:**
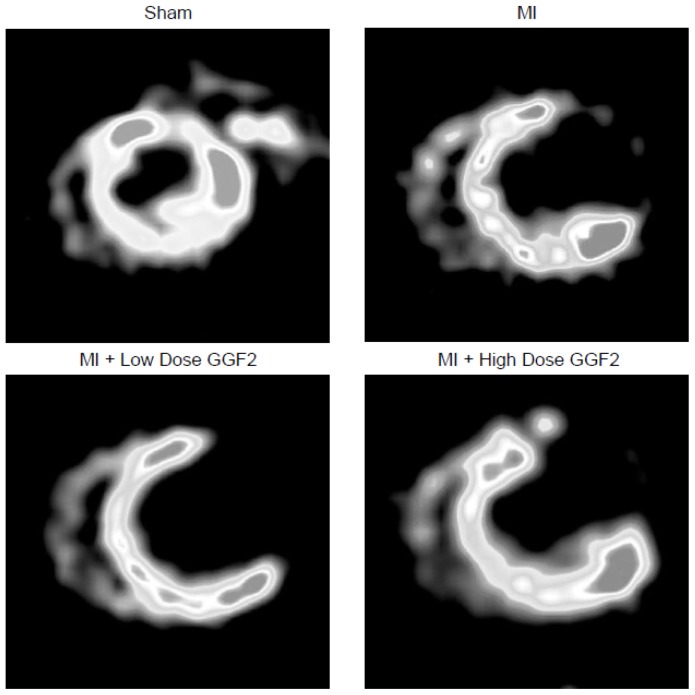
Effect of GGF2 treatment on myocardial PET glucose uptake. Short axis (transverse sections) images representative of sham, MI, and MI + GGF2 treated rats are displayed above. The sham rats showed prominent 18F-FDG uptake in all regions of the myocardium, whereas MI and MI + GGF2 treated rats show very little uptake in the infarct zone. Statistical analysis of cardiac standard uptake values was conducted by ANOVA, and results indicated that GGF2 treatment did not increase PET uptake in either remote or infarct regions (MI+LD, p = 0.79; MI+HD, p = 0.50).

Positron emission tomography (PET) was used to assess metabolic trends in post-MI rat myocardium treated with GGF2 both early and late after MI. Standard Uptake Values (SUVs) acquired by positron emission tomography were compared between all groups at the time points indicated above. In both infarct models, ^18^FDG uptake was similarly low in vehicle and GGF2-treated animal's infarct and remote regions (Figure 4). Improved LV function as a result of GGF2 treatment was not associated with a change in cardiac ^18^FDG uptake (MI+LD vs. Vehicle p = 0.79, MI+HD vs. Vehicle p = 0.50). In addition, there was no effect of GGF2 on the size of the infracted area.

### Myocardial ErbB Receptor Expression

**Figure 5:**
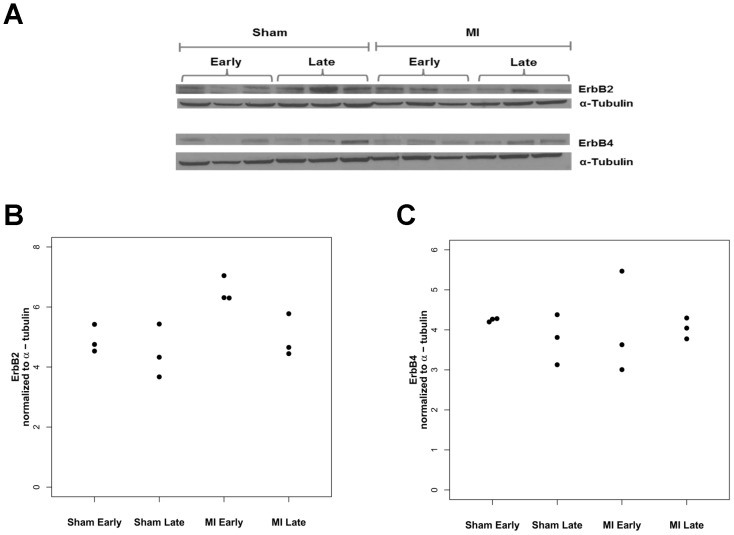
ErbB receptor protein expression in myocardium early and late after MI. A ) Representative Western blot for ErbB2 and ErbB4 in early post-MI (5 weeks) vs late post-MI (12 weeks). **B**) Quantification of myocardial ErbB2 receptor expression after densitometric analyses. ErbB2 expression was normalized to α-tubulin expression for each of the groups. ErbB2 expression was increased early post MI compared to sham (p = 0.01) and late post MI (p = 0.03). **C**) Quantificationof myocardial ErbB4 receptor expression after densitometric analyses of blotted bands. Normalized ErbB4 expression to α-tubulin was similar in all groups.

We examined whether changes in ErbB receptor expression, as observed in end-stage heart failure, could explain the differences in myocardial responsiveness to early versus late post-MI GGF2 treatment. Myocardial ErbB2 and ErbB4 receptor expression was analyzed from tissue lysates by immunoblot in rats in the groups treated with vehicle both early and late after MI (Figure 5). Myocardial ErbB2 but not ErbB4 receptor expression was greater in the early post-MI group compared to the sham as well as late post-MI rats.

### Effects of GGF2 Treatment on Cardiac Fibrosis and Oxidant Stress

Cardiac fibrosis was examined in formalin fixed sections of remote, non-infarcted myocardium from rats in the early post-MI experiment. Compared to the hearts of sham MI rats, the hearts from the MI rats treated with the vehicle demonstrated significantly greater fibrosis in LV myocardium remote from the site of infarction (p = 0.025) (data not shown). Treatment with low or high dose GGF2 did not alter the extent of myocardial fibrosis compared to vehicle-treated MI rats.

To determine whether GGF2 altered myocardial oxidative protein modification, post-MI heart homogenates were assayed for reactive carbonyl derivatives. While carbonylated protein content was more consistently low in the high dose GGF2-treated early-post MI group, there was wide variability in the sham operated and Vehicle-treated groups, and no significant difference was detectable by ANOVA (p = 0.62) (data not shown).

### Effects of GGF2 Treatment on Left Ventricular Gene Expression

**Figure 6:**
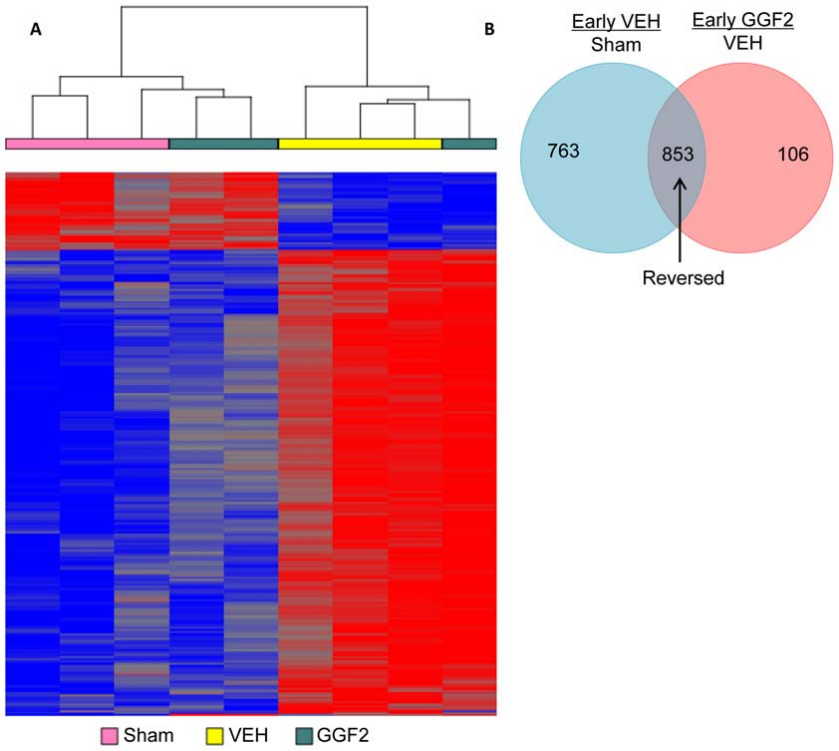
Effects of GGF2 treatment on gene expression early after MI. A) Hierarchical clustering of RNA normalized fluorescent signal values was performed for 1,720 genes determined to be differentially expressed (p value <0.05, FC >1.5) in LV samples collected 4 weeks post-MI. Color indicates relative levels of expression replicates, with bright red indicating the highest fluorescent signal values and bright blue representing the lowest in the various samples shown. Each row represents one probe set, found as up- or down-regulated in GGF2-treated rats at 4 weeks post-MI. Columns represent the three replicates for each group, color-coded by sample type (Sham: pink; vehicle: yellow; GGF2: green). Two prominently differential clusters were generated that separated Sham-operated rats from those that received vehicle treatment. Two of the GGF2-treated samples were more similar to Sham-operated controls, indicating a normalization of MI-induced gene expression for these two biological replicates. This pattern was not detected for the third GGF2-treated sample, which clustered with vehicle-treated rats. B) GGF2 treatment in the two biological replicate ‘responders’ showed reversal of 853 genes altered by MI, compared to Sham-operated rats.

In an effort to gain insight into the molecular mechanisms of GGF2-mediated restoration in cardiac function, we examined the effects of GGF2 treatment on the post-MI cardiac transcriptome. We chose post-MI rats given low dose GGF2 early after MI and included three biological replicates for the Sham-operated rats. This resulted in three biological replicates (Sham, VEH, and GGF2). Normalization followed by statistical analysis of the normalized hybridization signals resulted in 1,616 probe sets significantly altered (p value <0.05, fold-change at least 1.5) in untreated MI rats (VEH), compared to Sham-operated control animals (Figure 6, Table S1). As expected, many of these were genes known to be associated with MI (e.g. beta actin, cardiac muscle alpha actin, angiotensinogen, caveolin, collagens, myosin light and heavy chains, and myotilin).

For GGF2-treated compared to vehicle-treated MI rats, there were 959 significantly altered genes. The majority of these shared common functions, most notably genes encoding mitochondrial proteins essential for energy production. Of these 959 genes, 855 were altered in vehicle- treated MI rats compared to sham-operated controls as well as altered by GGF2 treatment compared to vehicle- treated MI. All but two of these 855 genes were normalized by GGF2 treatment (Figure 6). Hierarchical clustering of this normalized gene set showed that two of the GGF2-treated replicate rats were highly similar and clustered with the three Sham-operated replicate samples and apart from the vehicle-treated MI rats (Figure 6). One GGF2-treated sample appeared to be an outlier. Although we could not successfully identify the source of this variation, we cannot rule out a technical issue with this sample. However, it is also possible that the third GGF2-treated rat was a non-responder at the level of transcription, despite phenotypic similarities to the other GGF2-treated rats.

**Figure 7:**
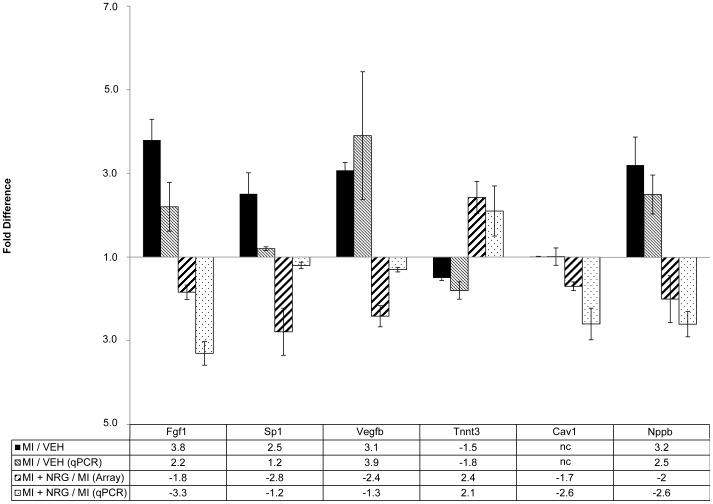
Real-time RT-PCR of eight genes in microarray data set. Eight genes were chosen for quantitative RT-PCR (qRT-PCR). Each bar represents one of four fold-differences obtained for vehicle-treated rats, compared to Sham-operated controls obtained using microarrays (black bars) or qRT-PCR (small, vertical striped bars) or GGF2-treated rats, compared to vehicle controls obtained using microarrays (large, vertical striped bars) or qRT-PCR (stippled white bars), as shown in the legend. Actual fold-changes are shown as a table, beneath each set of bars. Statistical analyses showed significant changes (n = 3 for each group, p<0.05) except where indicated (nc  =  no change).

The vast majority of the 853 normalized genes (787 genes) were up-regulated in response to induction of MI and down-regulated in GGF2-treated rats, compared to vehicle-treated controls. The remaining 66 genes were down-regulated in MI rats, compared to Sham-operated control animals, and induced in GGF2-treated rats. The inverse correlation between the entire set of 853 normalized genes was −0.87, based on Pearson's correlation coefficient, and the R-squared value was 0.92 when plotted and fit with a polynomial trend line. Functional analysis using Ingenuity Pathway Analysis software indicated that the most enriched functions for GGF2 normalized genes were energy production processes, including genes involved in the electron transport chain and metabolism related to production of energy precursors (Benjamini and Hochberg (B&H) corrected p values 5.8×10^−4^–1.1×10^−30^) (Table 2). Also significantly enriched were stress response and protein turnover, as well as organelle maintenance and cellular growth (B&H p values 1.1×10^−3^–7.4×10^−14^ and 1.3×10^−3^–7.4×10^−8^ respectively) (Table 2). Eight genes were chosen for validation by real-time RT-PCR, the results of which confirmed GGF2-mediated regulation of these genes (Figure 7).

**Table 2:** Functional Analysis of Altered Gene Expression in Post-MI Heart Muscle.

Over-represented Gene Ontology Category	MI-Veh vs. Sham	MI-GGF2 vs. MI-Veh
**Energy Production (B&H p values 5.8×10^−4^–1.1×10^−30^)**		
cellular respiration	35	26
electron transport chain	37	24
acetyl-CoA catabolic process	18	15
oxidation reduction	124	83
aerobic respiration	21	18
tricarboxylic acid cycle	18	15
fatty acid catabolic process	17	13
respiratory electron transport chain	15	9
ATP metabolic process	29	12
lipid oxidation	17	12
glycolysis	16	13
oxidative phosphorylation	26	11
generation of precursor metabolites and energy	94	62
**Metabolism Production (B&H p values 4.7×10^−4^–×10^−12^)**		
cellular macromolecule catabolic process	94	54
cofactor metabolic process	60	44
coenzyme metabolic process	52	40
cellular carbohydrate catabolic process	23	17
glucose catabolic process	20	14
cellular lipid catabolic process	25	18
**Stress Response/Protein Turnover (B&H p values 1.1×10^−3^–7.4×10^−14^)**		
response to oxidative stress	42	19
protein catabolic process	82	47
ubiquitin-dependent protein catabolic process	53	33
negative regulation of protein modification process	31	22
protein complex assembly and biogenesis	73	35
**Organelle Maintenance, Growth (B&H p values 1.3×10^−3^–7.4×10^−8^)**		
mitochondrion organization	40	29
ribonucleotide metabolic process	40	18
purine ribonucleotide metabolic process	39	18
RNA processing	71	31
nucleoside triphosphate metabolic process	36	15
macromolecular complex subunit organization	97	43
purine nucleotide metabolic process	45	23
mitotic cell cycle	48	26
macromolecular complex assembly	90	41
intracellular transport	79	37

*B&H* Benjamini and Hochberg multiple hypothesis correction. Numbers of genes found in each of the enriched functions are listed for the two comparisons shown. Functional categories are listed in descending order of significance. Complete list of genes can be found in Table S1.

### Effects of GGF2 Treatment on Left Ventricular Protein Expression

**Figure 8:**
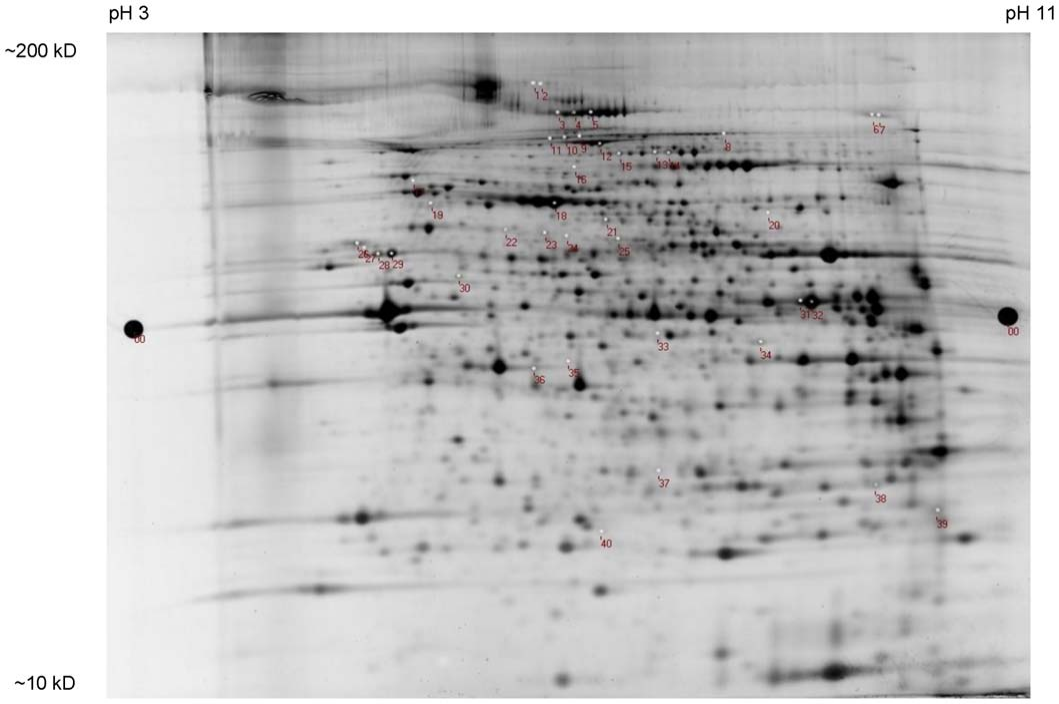
Examination of the effect of GGF2 on the myocardial proteome. Representative 2D gel image of total protein stain from the 5-gel coordinated DIGE experiment. A total of 300 µg of protein was loaded onto each DIGE gel. Altered proteins are indicated by numbers which correspond to line entries for identified proteins listed in Table 3.

In addition to whole transcriptome analysis, we examined the effects of low-dose GGF2 treatment early after MI on the post-MI cardiac proteome. A total protein stain of a representative gel is shown in Figure 8. Approximately 1,000 protein spots were visualized on each gel and protein spot patterns were essentially identical among the animals, as determined by spot matching and analysis using DeCyder software. A comparison of surviving LV myocardium from GGF2-treated post-MI rats (n = 4 individual animals) with vehicle-treated post-MI rats (n = 4 individual animals) and sham MI rats (n = 4 individual animals) revealed differential expression of 40 protein spots among the 3 groups that were identified by mass spectrometry and database interrogation as described in the Methods. The list was further filtered after focusing on proteins that showed altered expression in the MI group and whose altered expression was reversed by GGF2 treatment.

GGF2 treatment resulted in the down-regulation of several MI-induced proteins involved in metabolism and oxidative phosphorylation (Table 3). GGF2-induced proteins included glutathione peroxidase-3 (GPx-3) (#40), the expression of which was increased 37% (p = 0.023) in response to GGF2 treatment, as compared to vehicle-treated post-MI rats. Cardiac troponin I protein expression (#39) was down-regulated in the LV of vehicle-treated post-MI rats as compared to sham control rats. This effect was reversed by treatment with GGF2, as evidenced by a 48% increase in the expression of cardiac troponin I in post-MI rats that received GGF2 vs. vehicle (p = 0.025) (Table 3). This increase resulted in similar expression levels of cardiac troponin I between GGF2-treated post-MI hearts and sham MI hearts.

**Table 3:** Altered Protein Expression in Post-MI Heart Muscle in GGF2-treated Rats Relative to Vehicle Controls.

Protein	Gene	Function	MS Measurements					MI/Sham		HF		GGF2/VEH			
								This Study		Humans		This Study			
			Spot	pI	MW	Unique peptides	Spectral counts	MS	Array	Array	MS		Array		
***Energy Production, Metabolism, and Cellular Homeostasis***															
NADH-ubiquinone oxidoreductase 75 kDa subunit, mitochondrial+	Ndufs1	Electron transport	17	5.6	79	6	6	1.7	2.9	2.5		−1.6		−1.4	
Electron-transfer- flavoprotein, beta polypeptide+	Etfb	Electron transport	37	var	28	4	4	1.4	1.7	2.2		−1.4		−1.5	
Isocitrate dehydrogenase 3 (NAD+) beta+	Idh3b	TCA cycle	33	7.8	39	4	4	nd	2.9	1.7		−1.4		−1.6	
Enolase 3, beta, muscle	Eno3	Glycolysis, may play a role in muscle development and regeneration	35	7.1	47	7	8	nd	2.3	+/−		−1.3		−1.4	
Glycogen phosphorylase+	Pygm	Muscle enzyme involved in glycogenolysis	14	6.9	97	13	16	nd	1.8	−1.7		−1.3		−1.4	
Propionyl coenzyme A carboxylase, beta polypeptide	Ppcb	Lipid metabolism	24	7.2	58.6	3	4	1.4	1.5	nd		−1.3		−1.5	
Succinate dehydrogenase complex, subunit A, flavoprotein+	Sdha	Major catalytic component of mitochondrial respiratory chain	18	6.1	68	7	8	1.4	1.9	3.6		−1.2		−1.5	
Phosphoglycerate mutase 2 (muscle)	Pgam2	Glycolysis, striated muscle contraction, gluconeogenesis	37	var	28	4	5	1.4	2.4	nd		−1.4		−1.9	
3-hydroxybutyrate dehydrogenase, type 2+	Bdh2	Plays a key role in iron homeostasis and transport	37	var	28	3	3	1.4	1.8	1.8		−1.4		−1.5	
Polymerase I and transcript release factor^§^	Ptrf	Suggested to play an essential role in the formation of caveolae and the stabilization of caveolins, may also be involved in control of lipolysis	27	5.4	44	7	11	−1.3	−1.2	2		1.4		1.3	
Glutathione peroxidase 3^§^	Gpx3	Functions in the detoxification of hydrogen peroxide	40	6.4	23	3	3	nd	−1.3	−1.6		1.4		nd	
***Structural Proteins and Muscle Function/Development***															
Collagen, type I, alpha 1	Col1a1	Structural constituent of the extracellular matrix, focal adhesion	7	5.7	138	3	3	−1.6	−1.2	+/−		1.9		1.2	
Procollagen, type VI, alpha 3+	Col6a3	Muscle organ development, cell adhesion	1	8.4	288	18	18	−1.3	−1.5	−1.6		1.3		1.5	
Troponin I type 3 (cardiac)^ ‡^	Tnni3	Heart development, regulation of smooth muscle contraction and vasculogenesis	39	9.6	24	2	2	−1.4	nd	nd		1.5		nd	
Myosin binding protein C, cardiac	Mybpc3	Cardiac muscle contraction, cell adhesion	3	6.1	141	29	39	nd	nd	2.2		−1.5		nd	
Vimentin	Vim	Structural constituent of the cytoskeleton	26	5	54	11	13	nd	−1.3	nd		1.3		nd	
Guanine deaminase^‡^	Gda	Potentially involved in neuronal development	30	5.5	51	8	8	−1.3	1.2	nd		1.5		1.5/nd	
***Unknown***															
Inner membrane protein, mitochondria+	Immt	Unknown function, associates with Opa1 and Chchd3	17	5.4	82	3	3	1.7	1.7	1.5		−1.6		−1.4	
Coiled-coil-helix- coiled-coil-helix domain containing 3+	Chchd3	Mitochondrial protein, unknown function	37	var	28	3	3	1.4	2.7	2.2		−1.4		−1.6	

+ Supported by protein, gene expression, and human repository data; ^‡^supported by protein data only; conflicting results for protein and gene expression data ^§^; nd  =  no measurable difference; *var* varying; Fold Differences (FD) in protein expression were considered as ‘altered’ only if of a magnitude of at least 1.3 (or 30% increase/decrease) was detected and p value was <0.05, based on one-way ANOVA. Ndufs1 and Bdh2 were one of several possible proteins that could represent the two respective spots. These two proteins were chosen based on their dual expression change based on proteomics and microarray analysis. However, it is possible that one of the other potential proteins, which included myosin 6 and myosin binding protein C, were instead representative of the two significant differential spots.

We compared the proteomics results to our rat gene expression data and also to genes transcriptionally altered in failing human hearts. As shown in Table 3, we discovered nine rat proteins that were altered at the transcriptional and protein expression levels, in addition to matching gene transcription changes seen in failing human hearts. There were six additional proteins supported solely by gene expression and proteomics approaches. There were two proteins with expression changes that were discordant with the transcriptional changes.

## Discussion

The present study is one of a series that demonstrates that recombinant NRG-1β can improve cardiac function after myocardial infarction or other forms of cardiac injury in animals. The NRG-1β treatment effects were preserved in the setting of high fat diet, as well as later post-MI. GGF2 treated myocardium showed reversal of gene and protein expression changes induced by MI. The effects of GGF2 on myocardial gene and protein expression offer some possible explanations for the benefit of recombinant NRG-1β for CHF.

While the myocyte protective effects of recombinant NRG-1β are lost in the presence of increased levels of saturated fat *in vitro* [12], the beneficial effects of GGF2 on post-MI rat heart function was not altered by consumption of a high-fat diet. The high fat diet was initiated simultaneously with the initiation of GGF2, one week after induction of MI to avoid a confounding effect of diet on initial injury and scar formation. It is possible that longer duration exposure to high fat diet prior to GGF2 treatment would have yielded different results. However *in vitro*, small amounts of monounsaturated fat reverse the deleterious effects of palmitate on NRG-1β signaling and cell survival. Hence, the mix of fatty acids present in even the highest fat diets may be sufficient to prevent the disruption of NRG-1β/ErbB signaling.

It appears that the duration of heart failure after MI does alter sensitivity to GGF2. Increased myocardial ErbB2 expression early after MI may explain this result. Other investigators have shown that chronic heart failure is associated with reduced ErbB mRNA expression in both rodents and humans [29]. The mechanisms for increased ErbB2 expression observed warrant further investigation. Regardless of the mechanism, the variable responsiveness of chronic HF to recombinant NRG-1β should be considered in the development of a clinical research program for NRG-1β.

Bersell et al. have reported that injection of NRG-1β into post-MI mice for 12 weeks resulted in a decreased infarct size which coincided with improved myocardial function [33]. In the present study, we did not observe a change in infarct size as measured by PET imaging, although the duration of treatment was shorter than in their work. It is possible that with longer treatment there may be additional benefit of GGF2 through reduced infarct expansion.

The molecular pathways that mediate NRG-1β's effects on the heart remain unclear despite several studies demonstrating improved cardiac function. There are multiple cells in the heart that express ErbB receptors, respond to recombinant NRG, and could contribute to the observed improvement in cardiac function [30]. Microarray and proteomic analyses were used in this study to suggest possible pathways that might explain underlying molecular mechanisms responsible for GGF2′s effects. Analysis of un-treated post-MI rat hearts revealed up-regulation of genes and proteins that were mapped to energy production/metabolism and down-regulation of stress responses and proteins important for cardiac muscle structure and function. Treatment of post-MI rats with GGF2 reversed the majority of these alterations in gene and protein expression. These observations are in concert with recently reported findings that treatment of post-MI rats with a recombinant EGF-domain fragment of NRG-1β is associated with improved mitochondrial function in rodents [31]. Whether these responses are a direct result of NRG-1β treatment or an indirect consequence of improved cardiac function remains to be determined.

Other genes of note that were regulated by NRG-1β treatment in this and prior studies include *hopx* (homeodomain only protein x), a suppressor of serum response factor and GATA4, that regulates myocyte differentiation and cardiac hypertrophy. It is interesting that *hopx* expression is decreased in the NRG treated myocardium, opposite of what might be expected given reports of decreased *hoxp* gene expression in heart failure [32]. Also interesting is the induction of prothymosin alpha, an oncoprotein with established role in regulation of cell death in epithelial cells. While induction of myocyte division has been reported with NRG-1β treatment [33], there was minimal evidence for GGF2 altering expression of genes involved in cell cycle.

To assess the potential relevancy of this study's microarray results to human heart failure, the data were compared to raw data from eight studies representing 4 rat heart failure model studies and 4 human heart failure data sets obtained from online data repositories. The data were analyzed using the same methods employed to examine transcriptional changes in our rat model of MI, and genes altered at least 1.5-fold (p value <0.05) from all studies were compared (Table 4). There was considerable overlap between the current study and human heart failure, with 894 genes altered (same directionality) in both the rat model and human heart failure. Of note, 308 of these were normalized by GGF2 treatment in the current study.

**Table 4:** Comparison of GGF2 effects on gene expression to heart failure data sets.

Study	Group	n	# Genes altered	Overlap To Current Dataset	
				MI-Veh vs. Sham	MI-GGF2 vs. MI-Veh
***Rat Models***					
This Study	Sham	3			
	MI – Vehicle treated (v. Sham)	3	1,616	-	
	MI – GGF2 treated (v. MI + V)	2	959		-
GEO data (4 sets)	Sham	15			
	MI or IR	37	2,399	294	130
***Human Disease***					
C. Kirchoff Lab, Univ. Hospital Hamburg, E-TABM-480	Controls	4			
	Idiopathic	5	1,875	273	118
Cardiogenomics Lab, Harvard GSE1145	Normal	15			
	Cardiomyopathy	92	4,628	585	194
JM Hare Lab, Johns Hopkins, Internal Medicine, GSE1869	Unused Donor	6			
	Pre-LVAD Heart Failure	31	3,469	448	136
TP Cappola Lab, U Penn School of Medicine, GSE5406	Non-Failing	16			
	Cardiomyopathy	194	309	42	15
Total Across Human Studies		363	7,551	894	308

*MI* myocardial infarction; *IR* Ischemia-reperfusion; *n* number of subjects and/or biological replicates; *# Genes altered* number of genes significantly differentially expressed between disease groups and their respective controls; *Overlap* number of genes differentially expressed in the current study (either MI-Veh vs. sham or MI-GGF2 vs. MI-Veh) and the previously reported datasets. Complete list of genes can be found in Table S1.

We also compared our data to those of a previous study that assessed the effects of a NRG-1β fragment at 8 weeks post-MI in rats [4]. While the study design was significantly different than ours, there were nonetheless 24 genes altered in the same direction by NRG-1β treatment in both studies (Table 5). Several of these have known functions in metabolism. Notable non-metabolic genes have functions in transcriptional regulation, RNA splicing, and regulation of cell survival.

**Table 5:** Genes Similarly Altered by Treatment with GGF2 and EGF-domain only rhNRG in Post-MI Rats.

Gene Symbol	Name	GGF2	rhNRG	Function
Aldh6a1	Aldehyde dehydrogenase 6 family, member A1	−1.9	−1.4	Amino acid and lipid metabolism
Atp5g1	ATP synthase, H+ transporting, mitochondrial F0 complex, subunit C1	−1.9	−1.3	Energy production
Dcxr	Dicarbonyl L-xylulose reductase	−1.6	−1.4	Glucose metabolism
Dhrs7c	Dehydrogenase/reductase (SDR family) member 7C	−1.7	−1.3	Putative oxidoreductase
Eln	Elastin	1.6	1.2	Structural protein
Gdi1	GDP dissociation inhibitor 1	1.5	1.6	Small GTPase mediated signal transduction
Gstm7	Glutathione S-transferase, mu 7	−1.6	−1.4	Glutathione and xenobiotic metabolism
Hmgn1	High-mobility group nucleosome binding domain 1	1.6	2.1	Chromatin organization
Hopx	Homeodomain only protein x	−1.6	−1.3	Heart development, regulation of contraction
Macrod1	MACRO domain containing 1	−2.1	−1.3	Estrogen signaling, cell proliferation
Mut	Methylmalonyl-CoA mutase	−1.5	−1.3	Amino acid metabolism
Pgam2	Phosphoglycerate mutase 2 (muscle)	−1.9	−1.4	Gluconeogenesis, muscle contraction
Pnn	Pinin, desmosome associated protein	1.7	2.7	RNA processing and transport
Prpf39	PRP39 pre-mRNA processing factor 39 homolog	1.5	2.1	RNA processing and transport
Ptma	Prothymosin alpha	1.7	1.6	Anti-apoptosis
Rfk	Riboflavin kinase	−1.9	−1.3	Riboflavin metabolism
Snrp70	U1 small nuclear ribonucleoprotein polypeptide A	1.6	1.3	RNA splicing
Srrm2	similar to serine/arginine repetitive matrix 2	1.6	1.9	RNA splicing
Tada2b	Transcriptional adaptor 2 (ADA2 homolog, yeast)-beta	1.7	2.2	Transcriptional adaptor protein
Tst	Thiosulfate sulfurtransferase, mitochondrial	−1.5	−1.3	Mitochondrial rRNA import

Fold-changes in gene expression in treated compared to untreated animals are reported; negative value indicates downregulation in treated compared to controls.

In summary, we report that treatment with the GGF2 isoform of NRG-1β can improve LV function after MI in rats. The myocardial sensitivity to GGF2 is increased early compared to late after MI, and this correlates with increased myocardial ErbB2 receptor expression at the early time point. Furthermore, GGF2 treatment was associated with ‘normalization’ of the expression of many genes in the myocardium, providing directions to pursue in defining possible mechanisms of these effects. These results support further exploration of GGF2 as a therapeutic for systolic heart failure following MI.

## Supporting Information

Table S1 Complete list of genes altered by MI vs. Sham and/or GGF2 treatment.
(XLSX)
